# Patterns and predictors of osteoporosis medication discontinuation and switching among Medicare beneficiaries

**DOI:** 10.1186/1471-2474-15-112

**Published:** 2014-04-01

**Authors:** Huifeng Yun, Jeffrey R Curtis, Lingli Guo, Meredith Kilgore, Paul Muntner, Kenneth Saag, Robert Matthews, Michael Morrisey, Nicole C Wright, David J Becker, Elizabeth Delzell

**Affiliations:** 1 Department of Epidemiology, University of Alabama at Birmingham, Birmingham 35294, AL, USA; 2 Division of Clinical Immunology and Rheumatology, University of Alabama at Birmingham, Birmingham, AL, USA; 3 Department of Health Care Organization and Policy, University of Alabama at Birmingham, Birmingham, AL, USA

**Keywords:** Osteoporosis, Bisphosphonates, Medication switching, Discontinuation

## Abstract

**Background:**

Low adherence to bisphosphonate therapy is associated with increased fracture risk. Factors associated with discontinuation of osteoporosis medications have not been studied in-depth. This study assessed medication discontinuation and switching patterns among Medicare beneficiaries who were new users of bisphosphonates and evaluated factors possibly associated with discontinuation.

**Methods:**

We identified patients initiating bisphosphonate treatment using a 5% random sample of Medicare beneficiaries with at least 24 months of traditional fee-for-service and part D drug coverage from 2006 through 2009. We classified medication status at the end of follow-up as: continued original bisphosphonate, discontinued without switching or restarting, restarted the same drug after a treatment gap (≥ 90 days), or switched to another anti-osteoporosis medication. We conducted logistic regression analyses to identify baseline characteristics associated with discontinuation and a case-crossover analysis to identify factors that precipitate discontinuation.

**Results:**

Of 21,452 new users followed respectively for 12 months, 44% continued their original therapy, 36% discontinued without switching or restarting, 8% restarted the same drug after a gap greater than 90 days, and 11% switched to another anti-osteoporosis medication. Factors assessed during the 12-month period before initiation were weakly associated with discontinuation. Several Factors measured during follow-up were associated with discontinuation, including more physician visits, hospitalization, having a dual-energy X-ray absorptiometry test, higher Charlson comorbidity index scores, higher out-of-pocket drug payments, and upper gastrointestinal problems. Patterns were similar for 4,738 new users followed for 30 months.

**Conclusions:**

Among new bisphosphonates users, switching within and across drug classes and extended treatment gaps are common. Robust definitions and time-varying considerations should be considered to characterize medication discontinuation more accurately.

## Background

Adherence to oral bisphosphonate therapy has been reported to be suboptimal, with about 50% of patients who initiate treatment stopping within one year [1-3]. This poor adherence is thought to increase the risk of consequent morbidity (e.g., fractures) and associated costs [4]. Adherence to osteoporosis medications has been evaluated in many studies in the United States (US) [5-10], and certain factors inversely related to adherence have been identified, including drug related side effects, having multiple comorbid conditions, inconvenient dosing, higher cost, and hospitalization during the one year period before the initiation of osteoporosis medications. However, our understanding of factors related to poor adherence may be incomplete due to methods used to define discontinuation. Once patients discontinue their specific osteoporosis medication, some will switch to another anti-osteoporotic treatment. However, most previous research did not address patients’ switching agents, either to a different agent within the same therapeutic class, or to an agent in a different therapeutic class. One of the few analyses addressing osteoporosis medication discontinuation reported that 30% of patients resumed their medication within six months of discontinuation, and 48% resumed their medication within one year [4].

Hence, the commonly used methods of measuring adherence, which classify patients as discontinued after a gap of at least 90 days even if they later restart the same therapy [11,12], or exclude patients if they switch to another anti-osteoporosis medication or different dosage [13,14], may underestimate overall treatment adherence and hinder understanding of the utilization of bisphosphonate therapy. For a study of the efficacy, effectiveness, and/or safety of a specific type, route, or dose of bisphosphonate (e.g., alendronate, risedronate, ibandronate), a narrow definition of discontinuation may be appropriate. However, research on factors associated more generally with adherence, adverse effects or effectiveness of being treated with any anti-osteoporosis medication may benefit from a broader definition. In addition, factors precipitating the discontinuation of anti-osteoporosis medications have not been studied in-depth. In the osteoporosis field, these issues are of key importance given the growing interest in a ‘drug holiday’ [15,16] during which time patients are instructed to cease bisphosphonate therapy for a time (e.g., 1–3 years), making additional work on methods for discontinuation of anti-osteoporosis drugs important and timely. Accordingly, we conducted a study to describe discontinuation and switching patterns of anti-osteoporosis medications in the national US Medicare population, to evaluate factors associated with discontinuation using a variety of definitions, and to identify factors precipitating discontinuation of all anti-osteoporosis therapy.

## Methods

### Overview of design and data sources

To assess anti-osteoporosis medication utilization patterns and to identify baseline factors associated with alternative definitions of discontinuation, we conducted a retrospective cohort study. To identify factors precipitating discontinuation among adherent patients, we conducted a case-crossover analysis, nested within the cohort study [17]. Both the cohort and the case-crossover analyses used 2006–2009 data on a national 5% random sample of Medicare beneficiaries, obtained from the Centers for Medicare and Medicaid Services (CMS) Chronic Condition Data Warehouse [18]. For each beneficiary, data included information on demographic and insurance coverage; claims for inpatient, outpatient, skilled nursing facility, noninstitutional provider, home health, hospice, durable medical equipment services; and claims for prescription drugs. The institutional review board of the University of Alabama at Birmingham approved the study.

### Cohort study design

Eligible subjects for the cohort study were Medicare beneficiaries who were 65 years of age or older; lived in the US; were enrolled continuously in traditional Medicare fee-for-service and pharmacy coverage (Parts A, B and D) and not in a Medicare Advantage plan for at least 13 continuous months (12 months baseline and at least one month follow-up time); and were newly treated with a bisphosphonates. Bisphosphonate medications included infusion (IV) zoledronic acid, IV ibandronate, oral ibandronate, oral alendronate, or oral risedronate, during the period 2007 to 2009. We defined new treatment with a particular bisphosphonate as therapy initiated after a baseline period of 12 months during which no bisphosphonate prescription was filled and no administration of IV bisphosphonate occurred. We defined the “original” bisphosphonate as the one taken at initiation. We excluded patients who filled a prescription for raloxifene, calcitonin or teriparatide during baseline and for each beneficiary, follow-up began on the earliest treatment initiation date during the study period and ended on the earliest of first date of losing full Medicare coverage, the death date, or 12/31/2009.

**Figure 1:**
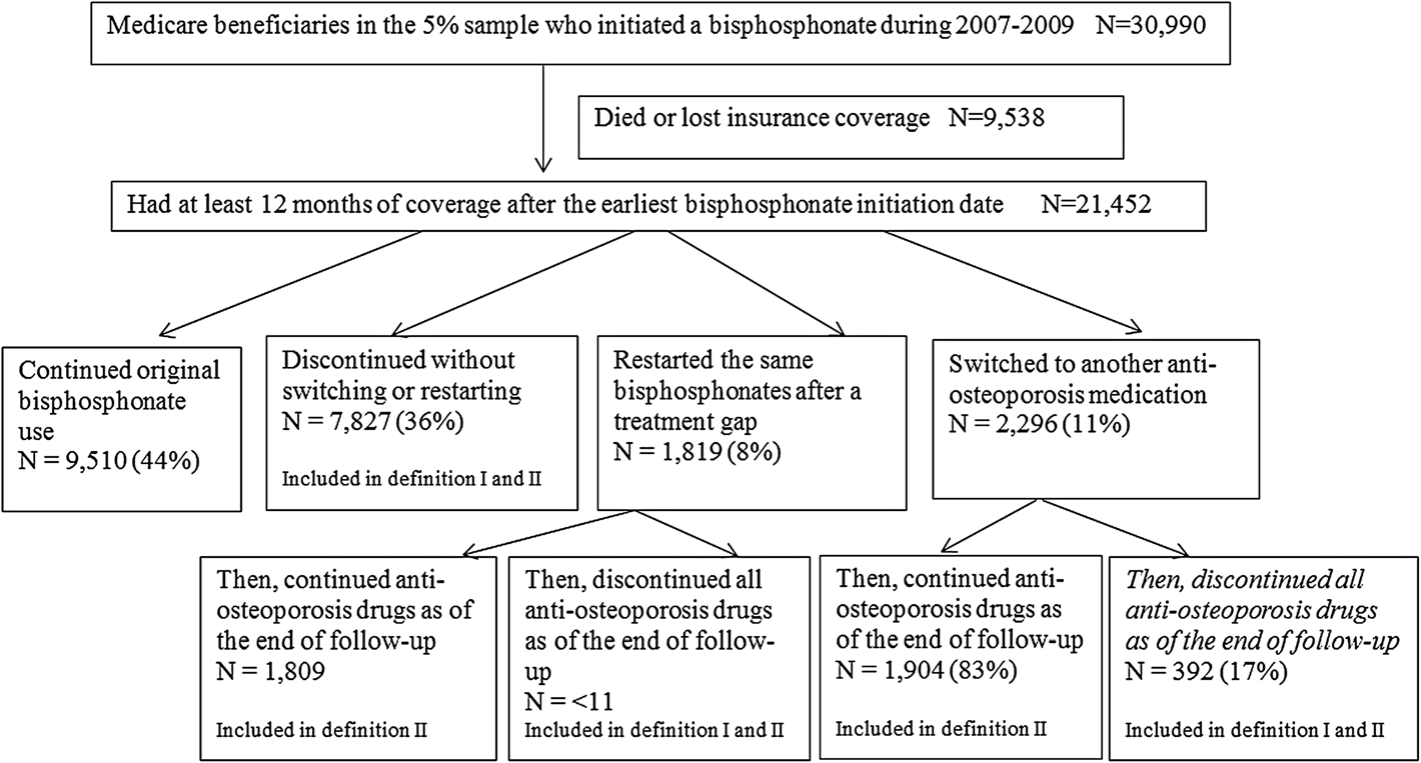
**Anti-osteoporotic medication discontinuation and switching among new bisphosphonate users at the end of 12 months of follow up. **Discontinuation definition I: Totally discontinued all anti-osteoporosis drugs as of the end of follow up. The total percentage of discontinuation I at the 12 months of follow-up is 38%. Discontinuation definition II: Totally discontinued all anti-osteoporosis drugs as of the end of follow up, or switched to another anti-osteoporosis medication, or stopped then restarted the same anti-osteoporosis drugs. The total percentage of discontinuation II at the 12 months of follow-up is 52%. As permitted N (CMS does not allow reporting of N < 11). Alendronate, risedronate, ibandronate, zoledronic acid, calcitonin, raloxifene, teriparatide were considered as different anti-osteoporosis medications. Branded and generic alendronates were considered as the same medication.

We examined utilization patterns during a follow-up period of 12 months, during which full Medicare coverage was required. We classified patients’ medication status at the end of 12 months of follow-up into four mutually exclusive groups: 1) continued original bisphosphonate use without a treatment gap, 2) discontinued without switching to a different osteoporosis medication or restarting the original bisphosphonate, 3) restarted the same bisphosphonate after a treatment gap or 4) switched to another drug (a different bisphosphonate, raloxifene, calcitonin or teriparatide). We defined a treatment gap as a period of at least 90 days, occurring after the end of the days supplied by a prescription or administration of a particular bisphosphonate drug and during which there was no further prescription fill or administration of the drug. We assigned days supplied as 90 days for IV ibandronate (administered at three-month intervals) and 365 days for IV zolendronic acid (administered annually). Based on the medication status at the end of 12 months of follow-up, we defined discontinuation using two definitions in the analysis evaluating the association between baseline characteristics and discontinuation of bisphosphonate therapy (Figure 1). Definition I classified a beneficiary as discontinued only if s/he had discontinued all anti-osteoporosis drugs as of the end of follow up. Definition II classified a beneficiary as discontinued if s/he had discontinued all anti-osteoporosis drugs as of the end of follow up, or switched to another type of bisphosphonate, or switched to a non-bisphosphonate anti-osteoporosis medication, or restarted the same anti-osteoporosis drug after a treatment gap of ≥ 90 days. Calcitonin, raloxifene, teriparatide were considered as different anti-osteoporotic medications during follow-up. For example, if a patient restarted the same bisphosphonate after a treatment gap, and then switched to calcitonin until the end of follow-up, we classified the patient as not discontinued in definition I, but discontinued in definition II. To assess longer-term adherence and switching, we conducted similar analyses among patients who had at least 30 months of follow-up, classifying their medication status at the end of 30 months of follow-up using the same definitions of discontinuation (Additional file 1).

### Case-crossover study design

The case-crossover design enables studying the association between transient exposure, and an acute event by comparing exposure in a period shortly before the event with the patient’s usual frequency of that exposure [19]. Because control information for each subject is based on his or her own past exposure experience in this design, results are not confounded by risk factors that are stable over time [19-21].

**Figure 2:**
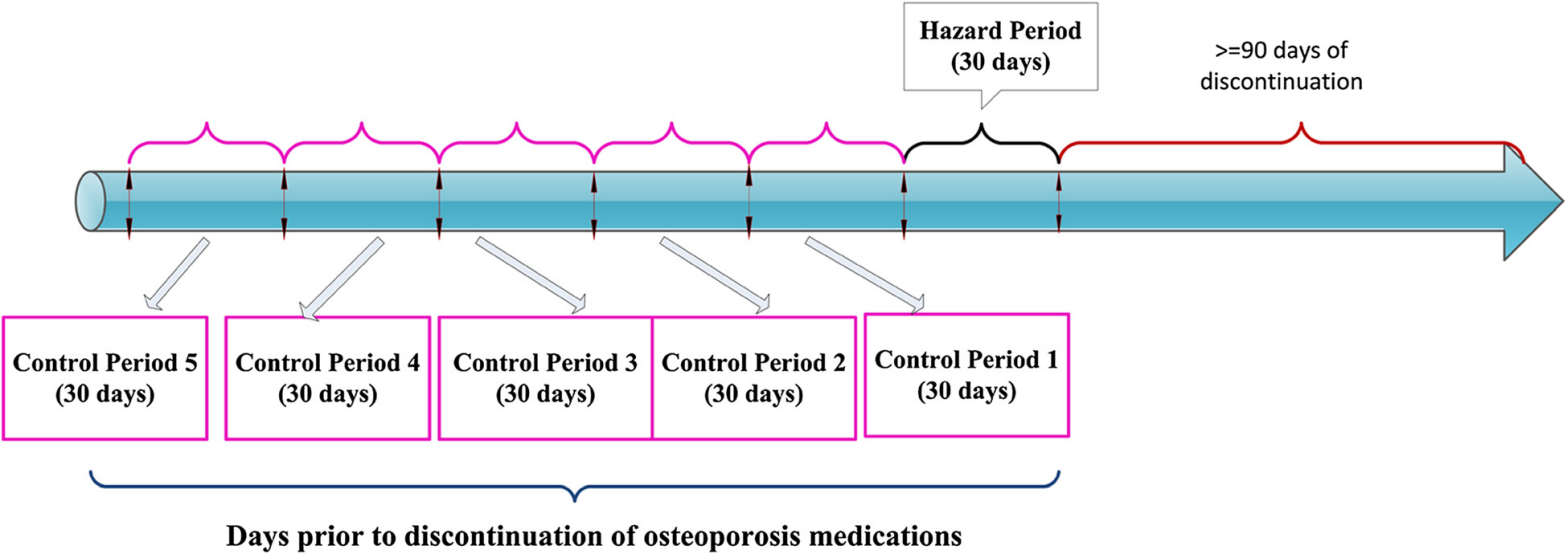
The case-crossover design of identifying factors precipitating discontinuation of osteoporosis medications.

To identify and quantify factors that may precipitate discontinuation to anti-osteoporosis medication, we evaluated each cohort member’s adherence to bisphosphonates during the first 12 months of treatment using the medication possession ratio (MPR), calculated as the total days of bisphosphonate exposure during the first 12 months of follow-up divided by 365 days, and then classified a subject as adherent to bisphosphonate therapy if s/he had an MPR ≥ 80%. The case-crossover analysis included subjects from the cohort study who were adherent to their original bisphosphonate during the first 12 months after treatment initiation, without switching, stopping and/or restarting their original bisphosphonate at any time during the first 12 months follow-up, who then discontinued their original bisphosphonate therapy (i.e., experienced a gap of 90 days or longer) during the subsequent follow-up (i.e., > 12 months after treatment initiation). We excluded those who had a hospitalization or skilled nursing home stay in the first 90 days after discontinuation in order to minimize misclassification, because claims data may not accurately measure drugs used during inpatient and skilled nursing facility stays (21). The follow-up period for identifying discontinuation began 12 months after initiation and continued until the earliest of the loss of full Medicare coverage, discontinuation, death, or 12/31/2009. For each eligible subject, we defined the “case” (or “hazard”) period as the 30 days immediately before bisphosphonate discontinuation, and we specified five “control” periods as the five 30-day periods immediately before the hazard period (Figure 2) [17,21,22].

### Factors possibly related to discontinuation

Factors measured during the baseline period for the cohort study and analyzed for possible association with different definitions of discontinuation included demographics, history of co-morbid conditions, history of preventive service and other medical service utilization, eligibility for a low income subsidy and use of prescription drugs other than anti-osteoporosis medications. Demographic characteristics included age, gender, race, geographic region and residential area-level income, all assessed as of the start of follow-up. We estimated income, as a proxy for socioeconomic status, using beneficiaries’ nine-digit ZIP codes linked to their 2000 Census block group of residence and the corresponding median household income [23]. We identified osteoporosis and history of fractures using International Classification of Disease (ICD)-9th revision diagnosis codes specific to the particular fracture sites from inpatient and physician encounter claims. ICD9 codes for osteoporosis included 733.0, 733.00, 733.01, 733.02, 733.03, 733.09, and for fracture included 800.xx-829.xx, and 733.1x. Other medical conditions, which may associated with fracture and consequently with anti-osteoporosis therapy patterns, included glucocorticoid-related and fall-related (predisposing to falls) conditions, diabetes, chronic kidney disease, depressive illness, acute myocardial infarction, other heart disease, metabolic bone disease and cancer [23]. We identified these conditions using the criterion of at least one diagnosis from inpatient or physician evaluation/management claims during baseline. Medications possibly associated with medication discontinuation or risk of fractures included anticonvulsants, antidepressants, antipsychotics, antihypertensives, lipid-lowering drugs, non-steroidal anti-inflammatory drugs, steroids, proton pump inhibitors, H2-receptor blockers, hormone replacement therapy, thiazolidinediones, and aromatase inhibitors. We identified these medications using the criterion of at least one claim for a prescription filled in Medicare Part D data during baseline.

Factors considered as possibly precipitating medication discontinuation in the case-crossover analysis (see detailed algorithm codes in Additional file 2) included (DXA) testing, skilled nursing home stays, hospitalization for any cause, entering the part D coverage gap [24], having a rheumatologist or endocrinologist visit, having an oncologist visit, eligibility for a low income subsidy, the occurrence of fracture, malignancy, upper gastrointestinal disease, adverse events (including osteonecrosis of the jaw, atrial fibrillation, esophageal cancer, renal disease, subtrochanteric or femoral shaft fractures), total number of physician visits, number of different medications, the Charlson comorbidity index [25], total Medicare costs and out-of-pocket drug costs. The first eight factors were dichotomous, whereas the rest had multiple categories based on their distribution during the last 30 days of the first 12 months of treatment.

For analyses evaluating the effect of baseline factors on discontinuation, the outcomes were the two alternative definitions of discontinuation at the end of 12 months following bisphosphonate initiation. For the analysis of factors precipitating discontinuation of anti-osteoporosis therapy, we selected as cases only those who totally discontinued bisphosphonate use at the end of follow-up.

### Analysis

We evaluated new bisphosphonate treatment patterns during 2007–2009 graphically for each bisphosphonate including branded alendronate without vitamin D, branded alendronate with vitamin D, generic alendronate, risedronate, oral ibandronate, IV ibandronate and IV zoledronic acid. We identified the number of new users of each specific bisphosphonate drug, calculated the proportion of each drug among all new users of bisphosphonates for each quarter of the study period, and calculated the proportion by medication status as of the end of 12 months of follow-up period.

We compared the baseline characteristics by medication status of new bisphosphonate users at the end of 12 months follow up and computed the standardized difference scores to evaluate the magnitude of differences between groups. We considered standardized difference scores that were less than 0.1 as clinically unimportant [26]. We conducted logistic regression analyses to identify baseline characteristics associated with the two different definitions of discontinuation at the end of 12 months of follow-up. Finally, we repeated all analyses to identify baseline characteristics associated with the two different definitions of discontinuation for patients who had at least 30 months of follow-up at the end of 30 months. These analyses used the odds ratio (OR) with its 95% confidence interval (CI) as the measure of association. Statistical significance was assessed at the alpha level of 0.05.

In the case-crossover analysis, we compared exposures in a subject’s hazard period (30 days immediately before discontinuation) and control periods (five 30-day periods immediately before the hazard period) [17,21,22]. In sensitivity analyses, we compared exposures in the 30 days immediately before discontinuation and four, three, two and one 30-day periods immediately before the hazard period respectively. We also carried out an additional sensitivity analysis in which the hazard and control time windows were extended to 60 days: that is, this analysis included one hazard period, consisting of the 60 days immediately before discontinuation, and two 60-day control periods immediately before the hazard period, so that the study period is as long as the main analysis We used conditional logistic regression models to compute ORs for the association between factors of interest and discontinuation for the case-crossover analysis. All statistical analyses were performed in SAS 9.3 (SAS institute).

## Results

**Figure 3:**
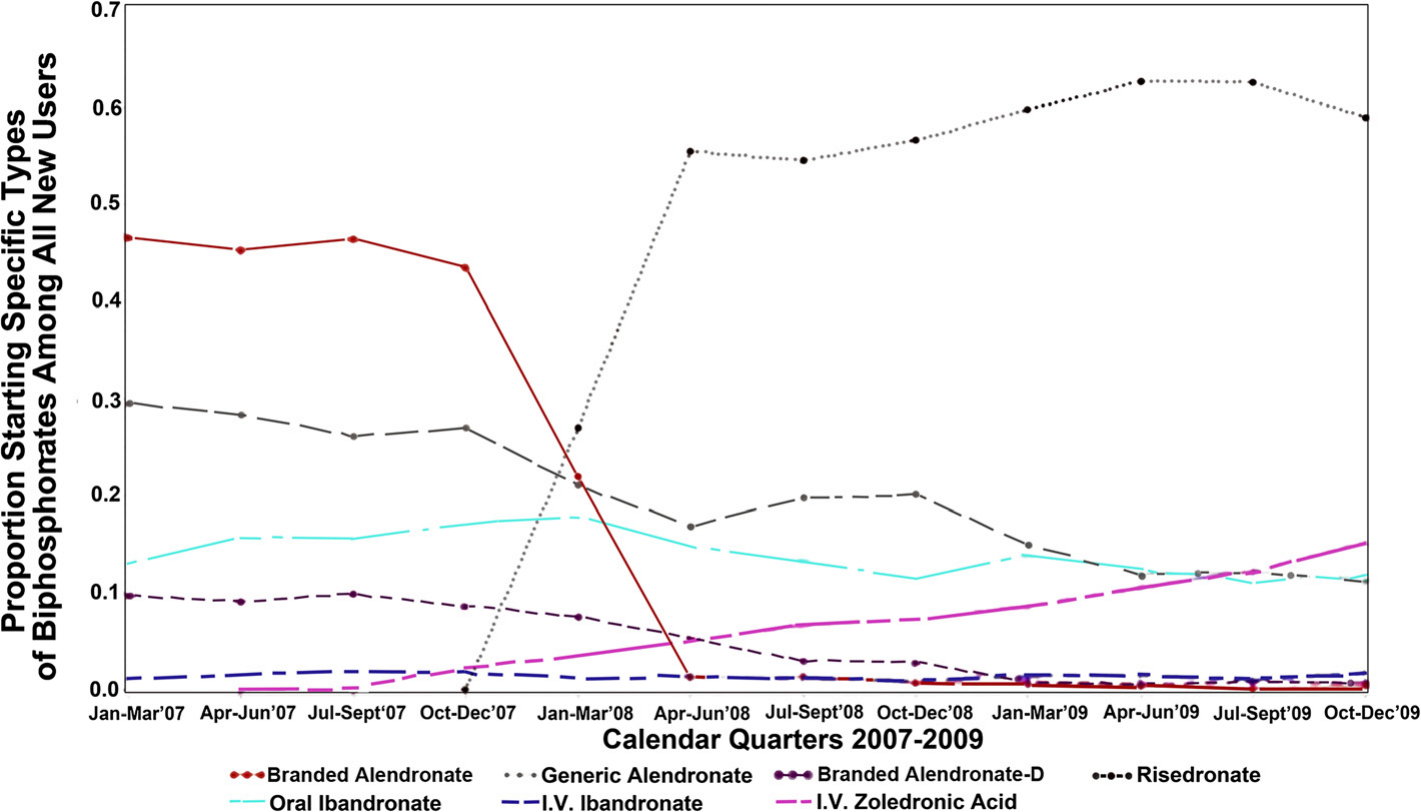
Proportion of Medicare beneficiaries starting specific types of bisphosphonates among all new users, by calendar quarter, 2007–2009.

We identified 30,990 Medicare beneficiaries in the 5% sample who initiated a bisphosphonate during 2007–2009 (Figure 3). The number of patients initiating each agent was 11,421 for generic alendronate, 6,173 for risedronate, 4,394 for oral ibandronate, 5,251 for branded alendronate without vitamin D, 1,794 for IV zoledronic acid, 1,532 for branded alendronate with vitamin D, and 425 for IV ibandronate. Overall, branded alendronate without vitamin D was the most commonly prescribed medication in 2007, whereas generic alendronate was the most commonly used drug in 2009.

Of the 30,990 new users of bisphosphonates identified in 2007–2009, 21,452 were eligible for inclusion in analyses of medication status at the end of 12 months of follow-up. (i.e., they had at least 12 months of coverage after the earliest treatment initiation date) (Figure 1). Of these new users followed for 12 months, 44% continued the same therapy, 36% totally discontinued without switching or restarting after a treatment gap, 8% restarted the same drug after a gap of greater than 90 days after the end of the days’ supply, and 11% switched to another bisphosphonate or non- bisphosphonate drug. By the end of 12 months of follow-up, most of beneficiaries who restarted the same drug after a treatment gap continued taking the anti-osteoporosis medications until the end of follow-up, whereas 17% of beneficiaries who switched to another drug discontinued all anti-osteoporosis medications. Of 4,738 eligible new users followed for 30 months, 19% continued the same therapy, 45% totally discontinued, 12% restarted the same drug after a gap, and 24% switched to another osteoporosis drug without a gap. By the end of 30 months of follow-up, 30% beneficiaries who switched to another drug discontinued all anti-osteoporosis medications (Additional file 1).

The standardized difference scores indicated that baseline characteristics of these four groups were statistically comparable except for history of hospitalization, residency in long-term care and history of DXA (Table 1). Compared to beneficiaries who discontinued without switching or restarting at the end of 12 months follow-up, beneficiaries who continued the original bisphosphonates were more likely to have undergone DXA testing and to have been residing in long-term care; beneficiaries who switched to another drug were more likely to have had a DXA and hospitalization during baseline.

**Table 1:** Baseline demographic and comorbidity characteristics by medication status of new bisphosphonate users at the end of 12 months follow up

**Medication status of new bisphosphonate users at the end of 12 months follow up**				
	**Continued original bisphosphonate use**	**Discontinued without switching or restarting**	**Restarted the same bisphosphonates after a treatment gap**	**Switched to another anti-osteoporosis medication**
**N**	9,510	7,827	1,819	2,296
**Age, years (SD)**	78 (6.8)	78 (6.8)	78 (6.7)	78 (6.8)
**Median Household Income, $ (SD)**	46,318 (22,808)	44,415 (22,715)	45,015 (23,123)	45,142 (22,540)
**Sex**				
Female	8,562 (90.3)	6,965 (89.0)	1,639 (90.1)	2,104 (91.6)
Male	948 (10.0)	862 (11.0)	180 (9.9)	192 (8.4)
**Race/ethnicity**				
Black	455 (4.8)	455 (5.8)	125 (6.9)	94 (4.1)
White	8,194 (86.2)	6,486 (82.9)	1,456 (80.0)*	1,920 (83.6)
Asian	360 (3.8)	319 (4.1)	92 (5.1)	130 (5.7)
Hispanic	285 (3.0)	382 (4.9)	95 (5.2)	107 (4.7)
Other	216 (2.3)	185 (2.4)	51 (2.8)	45 (2.0)
**Geographic region**				
Northeast	1,717 (18.1)	1,340 (17.1)	355 (19.5)	382 (16.6)
Midwest	2,534 (26.6)	1,805 (23.1)	417 (22.9)	568 (24.7)
South	3,540 (37.2)	3,287 (42.0)	701 (38.5)	903 (39.3)
West	1,719 (18.1)	1,395 (17.8)	346 (19.0)	443 (19.3)
**Charlson score**				
0	3,945 (41.5)	3,007 (38.4)	712 (39.1)	937 (40.8)
1-3	3,831 (40.3)	3,222 (42.2)	783 (43.0)	931 (40.5)
> 3	1,734 (18.2)	1,598 (20.4)	324 (17.8)	428 (18.6)
**Hospitalizations**	2,350 (24,7)	1,961 (25.1)	448 (24.6)	623 (27.1)*
**In long-term care**	1,146 (12.1)*	659 (8.4)	156 (8.6)	303 (13.2)
**History of any fractures**	1,051 (11.1)	704 (9.0)	162 (8.9)	272 (11.8)
**History of DXA**	5,641 (59.3)*	4,154 (53.1)	883 (48.5)	1,328 (57.8)
**Dual Eligible**	2,807 (29.5)	2,624 (33.5)	650 (35.7)	779 (33.9)
**Eligible for low income subsidy**	3,011 (31.7)	2,877 (36.8)	690 (37.9)	841 (36.6)
**Entered Medicare part D coverage gap**	1,826 (19.2)	1,613 (20.6)	367 (20.2)	471 (20.5)
**Proton pump inhibitors**	2,430 (25.6)	2,070 (26.5)	517 (28.4)	709 (30.9)
**Doctor visit type**				
Internal Medicine visits	6,220 (65.4)	5,085 (65.0)	1,170 (64.3)	1,564 (68.1)
Family Practice visits	4,446 (46.8)	3,654 (46.7)	781 (42.9)	1,107 (48.2)
Medical Oncology visits	940 (9.9)	721 (9.2)	159 (8.7)	222 (9.7)
Rheumatology/Endo visits	1,706 (17.9)	1,150 (14.7)	304 (16.7)	430 (18.7)*
**Number of Physician Visits**				
0-5	2,978 (31.3)	2,305 (29.4)	571 (31.4)	579 (25.2)
6-10	2,516 (26.5)	2,018 (25.8)	462 (25.4)	578 (25.2)
11-15	1,665 (17.5)	1,379 (17.6)	307 (16.9)	438 (19.1)
> 15	2,351 (24.7)	2,125 (27.1)	479 (26.3)	701 (30.5)

Numbers in table are n (column percent) or mean (standard deviation).

*Standardized difference score between current column and continued original bisphosphonate use column is greater than 0.1.

With 12 months of follow-up, factors associated with an increased ORs for total discontinuation of bisphosphonate therapy (definition I) included being male, being Hispanic versus non-Hispanic White, living in the South, having a Charlson score greater than zero, and having more than 10 physician visits during the baseline year (Table 2). Factors associated with a lower odds of total discontinuation included area income over $30,000, having a long-term care facility stay during baseline, having a DXA test during baseline, having rheumatologist or endocrinologist visit during baseline, having an oncologist visit during baseline, having history of osteoporosis and taking proton pump inhibitors during baseline All of these associations were weak, with positive associations having ORs below 1.5 and inverse associations having ORs of 0.8 or 0.9. Results were similar in the analysis that used discontinuation definition II after 12 months of follow-up and in analyses of both definitions of discontinuation after 30 months of follow-up (Additional file 3). The range of the c-statistics of the logistic regression models for these analyses was 0.58-0.61.

**Table 2:** Odds ratios (ORs) and 95% confidence intervals (CIs) for the association between baseline factors and discontinuation of bisphosphonate therapy at 12 months of follow-up based on discontinuation definition I during the period 2006–2009, cohort analyses

				**Discontinuation definition I**^**a**^
**Patient baseline characteristics**				**Adjusted**^**b **^**OR (95% CI)**
Sex	Male	vs	Female	1.1 (1.0-1.2)*
Race	Black	vs	White	1.1 (0.9-1.2)
	Asian	vs	White	0.9 (0.8-1.0)
	Hispanic	vs	White	1.1 (1.0-1.3)*
	Other	vs	White	1.0 (0.9-1.2)
Age	70-74	vs	65-69	1.0 (0.9-1.1)
	75-79	vs	65-69	1.0 (0.9-1.1)
	80-84	vs	65-69	1.0 (0.9-1.1)
	85plus	vs	65-69	1.0 (0.9-1.1)
Region	Midwest	vs	Northeast	0.9 (0.8-1.0)
	South	vs	Northeast	1.1 (1.0-1.2)*
	West	vs	Northeast	1.0 (0.9-1.1)
Area income 45000	30000-	vs	< 30000	0.9 (0.8-1.0)
60000	45000-	vs	< 30000	0.9 (0.8-0.9)*
75000	60000-	vs	< 30000	0.9 (0.8-0.9)*
	75000+	vs	< 30000	0.8 (0.8-0.9)*
Charlson score	1-2	vs	0	1.0 (0.9-1.1)
	> 2	vs	0	1.1 (1.0-1.2)*
Number of physician visits	6-10	vs	0-5	1.1 (1.0-1.2)
	11-15	vs	0-5	1.1 (1.0-1.2)*
	> 15	vs	0-5	1.3 (1.1-1.4)*
Hospitalization at baseline				1.0 (1.0-1.1)
Long-term care stay at baseline				0.6 (0.5-0.7)*
Fracture at baseline				0.9 (0.8-1.0)*
Dual-energy X-ray absorptiometry at baseline				0.8 (0.8-0.9)*
Internal medicine physician visit at baseline				1.0 (0.9-1.0)
Family practice physician visit at baseline				1.0 (0.9-1.1)
Oncologist visit at baseline				0.9 (0.8-1.0)*
Rheumatologist or endocrinologist visit at baseline				0.8 (0.7-0.9)*
Osteoporosis				0.8 (0.8-0.9)*
Proton pump inhibitors				0.8 (0.7-1.0)*

^a^Discontinuation definition I: Totally discontinued all anti-osteoporosis drugs as of the end of follow up.

^b^Adjusted for all factors listed in the table, and urban/rural residency, entering Medicare part D coverage gap at baseline, glucocorticoid-related and fall-related (predisposing to falls) conditions, diabetes, chronic kidney disease, depressive illness, acute myocardial infarction, other heart disease, metabolic bone disease, cancer, anticonvulsants, antidepressants, antipsychotics, antihypertensives, lipid-lowering drugs, non-steroidal anti-inflammatory drugs, steroids, H2-receptor blockers, hormone replacement therapy, thiazolidinediones, and aromatase inhibitors.

*p < =0.05.

Among subjects in the cohort study with high adherence (MPR ≥ 80%) in their first 12 months following bisphosphonate initiation, 922 patients subsequently discontinued their bisphosphonate therapy (Table 3). Factors, measured in the 30 days immediately preceding discontinuation, associated with discontinuation were having a DXA test, hospitalization, upper gastrointestinal symptoms or conditions, having more than four physician visits, Charlson score greater than 2, total out-of-pocket drug payments ≥ $20. Taking ≥ 3 different medications was inversely associated with discontinuation. The results of the sensitivity analyses were qualitatively similar to those of the main analysis, regardless of the number of control periods or the duration of the hazard and control periods (30 days v. 60 days) (data not shown).

**Table 3:** Odds ratios (ORs) and 95% confidence intervals (CIs) for the association between precipitating factors and discontinueation of bisphosphonate therapy, case-crossover analysis

**Factors potentially associated with discontinuation**^**a**^	**Univariate analysis**	**Multivariate analysis**
	**OR (95% CI)**	**Adjusted**^**b **^**OR (95% CI)**
Dual-energy X-ray absorptiometry	2.0 (1.3-3.1)	2.3 (1.4-3.6)
Any hospitalization	2.2 (1.5-3.1)	1.7 (1.1-2.7)
Fracture	0.7 (0.2-1.9)	0.4 (0.1-1.3)
Cancer	1.2 (0.7-1.9)	1.0 (0.6-1.8)
Entering Medicare part D coverage gap	1.0 (0.8-1.3)	0.9 (0.7-1.3)
Adverse effects	1.6 (1.1-2.3)	1.0 (0.7-1.6)
Skilled nursing home	0.7 (0.2-2.1)	0.4 (0.1-1.4)
Rheumatologist or endocrinologist visit	1.1 (0.7-1.7)	0.8 (0.5-1.4)
Upper gastrointestinal disease	3.2 (1.4-7.3)	3.4 (1.5-7.7)
Eligible for low income subsidy	4.9 (0.8-28.7)	5.7 (1.0-32.4)
Number of ambulatory physician visits during each 30 day period		
0	Ref (1.0)	Ref (1.0)
1-4	1.2 (1.0-1.4)	1.2 (1.0-1.5)
> 4	2.4 (1.4-4.1)	2.5 (1.3-4.5)
Number of medications during each 30 day period		
0-2	Ref (1.0)	Ref (1.0)
3-5	0.8 (0.7-1.0)	0.5 (0.4-0.6)
> 5	0.5 (0.4-0.7)	0.3 (0.2-0.4)
Charlson score during each 30 day period		
0	Ref (1.0)	Ref (1.0)
1-2	1.3 (0.8-1.9)	1.4 (0.9-2.1)
> 2	1.7 (1.0-3.1)	2.5 (1.3-4.7)
Total Medicare cost during each 30-day period		
$ 0-90	Ref (1.0)	Ref (1.0)
$ 91-440	0.9 (0.7-1.1)	0.9 (0.7-1.1)
$ > 440	1.0 (0.8-1.3)	0.9 (0.8-1.3)
Out-of-pocket drug payments during each 30 day period		
$ 0-19	Ref (1.0)	Ref (1.0)
$ 20-84	1.6 (1.2-2.0)	1.6 (1.3-2.1)
$ > 84	5.4 (4.4-6.7)	6.6 (5.3-8.1)

^a^Hazard period is defined as 30 days immediately before discontinuation. Control periods are defined as five 30-day periods immediately before the hazard period.

^b^Adjusted all the factors listed in the table.

## Discussion

We found large temporal changes in the pattern of use of several types of bisphosphonates in 2007–2009 among Medicare beneficiaries. Our analysis of factors associated with different definitions of discontinuation found factors measured at baseline were, at most, weakly associated with discontinuation and differed little according to the definitions of discontinuation. However, in the case-crossover analysis evaluating the effect of factors measured immediately before discontinuation, a DXA test, having upper gastrointestinal problems, having more physician visits, higher Charlson comorbidity scores, and higher out-of-pocket drug payments, were associated with total discontinuation of bisphosphonate therapy after one year of high adherence, whereas a higher total number of all types medications dispensed was associated inversely with discontinuation.

Starting in 2008, we found the major increases in bisphosphonate utilization were for generic alendronate and IV zoledronic acid, and the major decreases were for branded alendronate products and risedronate. Our findings are consistent with a report from Medco Health Solutions, Inc (a healthcare company) indicating that branded alendronate lost 84% of its market share from retail stores and 94% of its market share from mail pharmacies during the first 30 days after the launch of generic products [27]. Several factors could explain the increase in IV/injection bisphosphonates utilization that we observed during the 2007–2009 time period. More aggressive marketing and perceptions of potentially better adherence with IV compared to oral bisphosphonates could lead to greater prescribing of IV therapies. Parenteral administration also may minimize certain side effects, such as gastrointestinal upset, and avoids the complex instructions required for proper administration of oral bisphosphonates [28].

We also found that switching within the bisphosphonates class, switching out of the bisphosphonate treatment class, and having treatment gaps were common among new bisphosphonate users. For our purposes, discontinuation was defined in two alternative ways: first, as total discontinuation of any osteoporosis medications; second, as total discontinuation, having a therapy gap then restarting the same bisphosphonate, or switching to another therapy during follow-up. Compared to the strict definition (I) of discontinuation, an additional 17-29% of people were classified as discontinued due to switching to other types of bisphosphonate or to non-bisphosphonate osteoporosis medications or due to a gap in therapy with a particular type of bisphosphonate.

Our findings are consistent with those of prior studies showing that people are prone to switch or restart bisphosphonates after discontinuation [4,29]. Using 1996–2002 Pharmaceutical Assistance Contract for the Elderly (PACE) of Pennsylvania data, Brookhart et al. reported that among patients who stopped bisphosphonate therapy for at least 60 days, an estimated 30% restarted treatment within six months [4]. Their results are higher than the percentage restarting in our study, possibly because the prior analysis examined the therapeutic class of all bisphosphonates combined, while our analysis examined each form of bisphosphonate separately. Brookhart et al. also defined a gap as 60 days, whereas we used a gap length of 90 days. Another study, in a commercial health plan population during the period of 2006–2008, reported substantial discontinuation, restarting, and switching among new users of various anti-osteoporosis medications [29]. For example, 52-67% of new users of specific types of bisphosphonates discontinued their original medication within 12 months, 13-22% restarted their original medication after a greater than 90-day treatment gap, and 0.4-9.6% switched to another drug. Therefore, examining patterns of restarting and switching in future osteoporosis studies may generate a more complete definition and assessment of adherence, particularly in studies that include time periods when a major new anti-osteoporosis medication, such as generic alendronate, has been introduced.

Regardless of length of follow-up or the definition of discontinuation, the factors that we assessed at baseline displayed generally weak associations or no relation with discontinuation. With area under the receiver operator curve as low as 0.58-0.60 in both multivariable logistic models, these results are consistent with those of prior studies reporting that baseline characteristics, measured only at treatment initiation, yielded a poor ability to discriminate osteoporosis patients with high adherence versus low adherence [30].

Our case-crossover analysis was designed to identify factors emerging during follow-up that lead to discontinuation among adherent bisphosphonate users. The results indicated that events occurring during follow-up are likely to be more important as predictors of adherence and persistence than are characteristics measured at therapy initiation. Specifically, we identified six important factors that were positively associated with discontinuation – DXA, more than four ambulatory physician visits, hospitalization, upper gastrointestinal disorders, higher Charlson comorbidity index, and higher out-of-pocket drug payments.

DXA is the gold standard for diagnosing osteoporosis, and it may play a role in monitoring response to osteoporosis treatment [31]. Our finding that after one year of high adherence, having a DXA was significantly associated with discontinuation, suggests that physicians and patients may consider discontinuing osteoporosis medications if DXA test results indicate a bone mineral density improvement treatment [32] or that patients may discontinue the therapy due to a lack of improvement in bone mineral density [33]. Unfortunately Medicare data do not include DXA test results, which would be required to confirm this interpretation. Our observation that having more than four ambulatory physician visits was strongly associated with discontinuation contrasts with another study that reported that physician visits were related to patients’ reinitiating therapy [4]. The discordant results may reflect differences in study populations: our case-crossover analysis was limited to people who had been adherent for at least one year and then experienced discontinuation, whereas the latter study was limited to new users. Also, it is possible that patients with more diseases tend to see physicians more often and stop taking drugs with fewer perceived beneficial effects [34,35].

Our study confirms results from other investigations reporting that upper gastrointestinal conditions are significantly associated with bisphosphonate discontinuation [1,11]. Finally, our findings indicate that patients who have more comorbid conditions, pay more out-of-pocket for drugs, or stay in the hospital are much more likely to discontinue drug therapy. These associations are consistent with the phenomenon called the “sick stopper”, wherein patients who are getting sicker and spending more healthcare dollars maybe more likely to discontinue treatments for non-symptomatic conditions, such as osteoporosis [35].

A major strength of our study was its assessment of factors possibly associated with discontinuation both at baseline and, in the case-crossover analysis, during follow-up. Other strengths include the study’s large size, generalizability to the entire US traditional fee-for-service Medicare population with Part D coverage, the use of prescription claims to define exposures instead of self-reported drug usage, and our sensitivity analyses. The methodological aspects to our work should grow in importance given increasing interest on drug holidays from bisphosphonate exposure. We varied the definition of discontinuation across a range of possible options and examined factors associated with discontinuation, which is expected to be useful for future analyses that examine new reasons for patients to stop therapy such as a drug holidays [15,16].

The main limitation was the study’s reliance on claims data to assess utilization and possible reasons for discontinuation. Claims data do not includes baseline or follow-up bone mineral density measures, which could have influenced the likelihood of adherence. Our case-crossover analysis was limited to patients who had been adherent to bisphosphonates for 12 months, whereas the cohort analysis used new users, therefore, the characteristics of subjects discontinuing included in the case-crossover analysis were not comparable to those totally discontinued in the cohort analysis. There also could be misclassification of safety outcomes without well validated claims-based algorithms (e.g., osteonecrosis of the jaw, atypical fracture, and upper gastrointestinal symptoms). The possibility that some patients may have filled their anti-osteoporosis prescriptions without submitting a claim to Medicare is another potential limitation. However, we recently reported that this may occur for a maximum of 15% of generic prescriptions, using alendronate as an example, available through low-cost pharmacy programs [36].

Finally, our analyses included patients who had primary adherence (i.e., they filled a prescription for a bisphosphonate), but not patients with primary non-adherence (i.e., had anti-osteoporosis prescriptions but never have it filled or administrated), and our case-crossover analysis was further restricted to patients who were adherent for 12 months after initiation of therapy. Thus, the results may not be generalizable to all Medicare patients prescribed and taking bisphosphonate drugs.

## Conclusions

Generic alendronate and IV zoledronic use among Medicare beneficiaries increased substantially during 2007–2009. Among new bisphosphonate users, switching among bisphosphonates, switching out of the bisphosphonate therapeutic class, and having treatment gaps were common. Factors measured at therapy initiation were at most weakly associated with discontinuation, regardless of varying the definition of discontinuation and the length of follow-up. In contrast, several factors measured during follow-up appeared to precipitate discontinuation within 12 months of initiating bisphosphonate therapy including DXA, more than six ambulatory physician visits, hospitalization, upper gastrointestinal disorders, higher Charlson comorbidity score, and higher out-of-pocket drug payments. Further studies are needed to characterize medication discontinuation more carefully with consideration of time-varying factors.

## Abbreviations

CI: Confidence interval; CMS: Centers for medicare and medicaid services; DXA: Dual-energy X-ray absorptiometry; ICD: International classification of disease; IV: Infusion; MPR: Medication possession ratio; OR: Odds ratio; PACE: Assistance contract for the elderly; US: United States.

## Authors’ contributions

Conception and design: HY, ED, KS, MK, PM, JRC. Acquisition of Data: HY, ED, KS, MK, MM, JRC. Analysis and interpretation of data: HY, ED, KS, MK, PM, MM, NCW, LG, RM, DJB, JRC. Drafting manuscript: HY. Critical revision of manuscript for important intellectual content: HY, JRC, KS, MK, MM, NCW, DJB, ED. Statistical analysis: HY, RM, LG. Obtaining Funding: JRC, KS, MK, MM, ED. Administrative, technical, or material support: RM, S, KS, MK, MM, ED, JRC. Study supervision: ED, KS, JRC. All authors read and approved the final manuscript.

## Pre-publication history

The pre-publication history for this paper can be accessed here:

http://www.biomedcentral.com/1471-2474/15/112/prepub

## Supplementary Material

Additional file 1 Anti-osteoporotic medication use of new bisphosphonate users at the end of 30 months of follow up. Click here for file

Additional file 2 **“Algorithms for identifying the possible trigger factors in the hazard and control period”.** In the submitted version, it was in the first row of the table. Click here for file

Additional file 3 Baseline factors associated with discontinuation of bisphosphonate therapy at 30 of follow-up, based on discontinuation definition I during the period 2006-2009, cohort analyses. Click here for file
