# Supplementary material for: Patterns and predictors of osteoporosis medication discontinuation and switching among Medicare beneficiaries
Source: BMC Musculoskelet Disord. 2014 Apr 1;15:112. doi: 10.1186/1471-2474-15-112 (PMC4022369; doi:10.1186/1471-2474-15-112)
Supplement: Additional file 1 — Anti-osteoporotic medication use of new bisphosphonate users at the end of 30 months of follow up. [file 1471-2474-15-112-S1.docx]

Additional file **1**: Anti-osteoporotic medication use of new bisphosphonate users at the end of 30 months of follow up

Medicare beneficiaries in the 5% sample who initiated a bisphosphonate during 2007-2009 N=30,990

Medicare beneficiaries in the 5% sample who initiated a bisphosphonate during 2007-2009 N=30,990

Died or lost insurance coverage within 30 months follow-up N=26,252

Had at least 30 months of coverage after the earliest bisphosphonate initiation date N=4,738

Continued original bisphosphonate use

N = 910 (19%)

Discontinued without switching or restarting

N = 2,123 (45%)

Included in definition I and II

Restarted the same bisphosphonates after a treatment gap

N = 583 (12%)

Switched to another anti-osteoporosis medication

N = 1,122 (24%)

*Discontinued all anti-osteoporosis drugs as of the end of follow-up*

N = <11

Included in definition I and II

*Continued anti-osteoporosis drugs as of the end of follow-up*

N = 580

Included in definition II

*Discontinued all anti-osteoporosis drugs as of the end of follow-up*

N = 339 (30%)

Included in definition I and II

*Continued anti-osteoporosis drugs as of the end of follow-up*

N = 783 (70%)

Included in definition II

**Discontinuation definition I: Totally discontinued all anti-osteoporosis drugs as of the end of follow up. The total percentage of discontinuation I at the 30 months of follow-up is 56%.**

**Discontinuation definition II: Totally discontinued all anti-osteoporosis drugs as of the end of follow up, or switched to another anti-osteoporosis medication, or stopped then restarted the same anti-osteoporosis drugs. The total percentage of discontinuation II at the 30 months of follow-up is 81%.**

**† As permitted N (CMS does not allow reporting of N<11)**

**^a^Alendronate, risedronate, ibandronate, zoledronic acid, calcitonin, raloxifene, teriparatide were considered as different anti-osteoporosis medications. Branded and generic alendronates were considered as the same medication.**
