# Supplementary material for: Patterns and predictors of osteoporosis medication discontinuation and switching among Medicare beneficiaries
Source: BMC Musculoskelet Disord. 2014 Apr 1;15:112. doi: 10.1186/1471-2474-15-112 (PMC4022369; doi:10.1186/1471-2474-15-112)
Supplement: Additional file 2 — “Algorithms for identifying the possible trigger factors in the hazard and control period”. In the submitted version, it was in the first row of the table. [file 1471-2474-15-112-S2.docx]

**Additional file 2**

| **Algorithms for identifying the possible trigger factors in the hazard and control period.** | |
| --- | --- |
| **Factors** | **Definition** |
| Dual-energy X-ray absorptiometry (DXA) testing | Any claims from inpatient, outpatient, physician and durable medical equipment service:  ICD-9 procedure codes: 88.98  HCPCS codes: 76070, 76071, 76078, 78350, 78351, 76977, 76075, 76076, 76077, G0130, 77080, 77081, 77082, 77083, 77078, 77079 |
| Skilled nursing home stays | Any days of skilled nursing home stay from skilled nursing facility file |
| Hospitalization for any cause | Any days of hospitalization from inpatient file |
| entering the part D coverage gap | Any claims from prescription drug claims containing: benefit phase: II, PI, DI |
| having a rheumatologist or endocrinologist visit | Any claims from physician and durable medical equipment service containing the following codes: Provider specialty: 46, 66 |
| eligibility for a low income subsidy | Identified for Part D denominator file if cost sharing group contain  01,02,03,04,05,06,07,08 |
| The occurrence of fracture | ICD-9 diagnosis codes included 800.xx-829.xx, 733.1x; |
| The occurrence of malignancy | ICD-9 diagnosis codes included 140.xx-208.xx, but not 173.xx |
| The occurrence of upper gastrointestinal disease | ICD-9 diagnosis codes included 5310,5311,5312,5314,5315,5316,5320,5321,5322,5324,5325,5326,5340,5341,5342,5434,5345,5346,5330,5331,5332,5334,5335,5336,5780,5781,5789 |
| adverse events (including osteonecrosis of the jaw, atrial fibrillation, esophageal cancer, renal disease, subtrochanteric or femoral shaft fractures), | ICD-9 codes for ONJ included 526.4, 522.7, 526.5, 733.45 paired with physician evaluation and management codes  ICD-9 codes for atrial fibrillation included 427 paired with physician evaluation and management codes  ICD-9 codes for esophageal cancer included 150 paired with physician evaluation and management codes  ICD-9 codes for renal disease included 527.4, 580.xx, 584, 584.5, 584.6, 584.7, 584.8, 584.9, 586, 788.5, 791.2, 791.3, 016.0, 042.9, 095.4, 189.x, 202.89, 223.x, 236.91, 250.4x, 274.1, 403.x, 404.x, 405.x, 440.1, 442.1, 447.3, 453.3, 572.4, 581, 582, 583, 585.x, 586, 587, 588, 589, 593, 599.7, 753.x, 791.0, 794.4, 866.xx, 984.9, 996.7, 997.72, V42.0, 996.81, paired with physician evaluation and management codes  ICD-9 codes for subtrochanteric or femoral shaft fractures included 820.22, 821.00, 821.01 paired with physician evaluation and management codes, but no concurrent major trauma E-codes. |
| Total number of physician visits | All types of physician visits from physician file. Cutoff points: 0, 1-4, >4 |
| The Charlson comorbidity score | Cutoff points: 0, 1-2, >2 |
| Total Medicare drug costs | Cutoff points: $ 0-90, $ 91-440, $ >440 |
| Out-of-pocket drug costs | Cutoff points: $ 0-19, $ 20-84, $ >84 |
