# Supplementary material for: Patterns and predictors of osteoporosis medication discontinuation and switching among Medicare beneficiaries
Source: BMC Musculoskelet Disord. 2014 Apr 1;15:112. doi: 10.1186/1471-2474-15-112 (PMC4022369; doi:10.1186/1471-2474-15-112)
Supplement: Additional file 3 — Baseline factors associated with discontinuation of bisphosphonate therapy at 30 of follow-up, based on discontinuation definition I during the period 2006-2009, cohort analyses. [file 1471-2474-15-112-S3.docx]

**Additional file 3:** Baseline factors associated with discontinuation of bisphosphonate therapy at 30 of follow-up, based on discontinuation definition I during the period 2006-2009, cohort analyses.

|  | | | **Discontinuation Definition I^a^** |
| --- | --- | --- | --- |
| **Patient baseline characteristics** | | | **Adjusted^b^ OR (95% CI)** |
| **Sex male** | **vs** | **female** | ***1.3(1.1-1.6)**** |
| **Race black** | **vs** | **white** | ***1.0(0.8-1.4)*** |
| **asian** | **vs** | **white** | ***0.7(0.5-0.9)**** |
| **hispanic** | **vs** | **white** | ***0.8(0.5-1.3)*** |
| **other** | **vs** | **white** | ***0.8(0.5-1.3)*** |
| **Age 70-74** | **vs** | **65-69** | ***0.8(0.7-1.0)*** |
| **75-79** | **vs** | **65-69** | ***0.8(0.7-1.0)*** |
| **80-84** | **vs** | **65-69** | ***0.9(0.7-1.1)*** |
| **85plus** | **vs** | **65-69** | ***1.1(0.9-1.4)*** |
| **Region midwest** | **vs** | **northeast** | ***0.8(0.7-1.0)**** |
| **south** | **vs** | **northeast** | ***1.2(0.9-1.4)*** |
| **west** | **vs** | **northeast** | ***0.9(0.7-1.1)*** |
| **Area income 30000-45000** | **vs** | **<30000** | ***0.8(0.7-1.0)**** |
| **45000-60000** | **vs** | **<30000** | ***0.9(0.7-1.1)*** |
| **60000-75000** | **vs** | **<30000** | ***1.0(0.8-1.3)*** |
| **75000+** | **vs** | **<30000** | ***0.8(0.7-1.1)*** |
| **Charlson score 1-2** | **vs** | **0** | ***1.1(0.9-1.3)*** |
| **>2** | **vs** | **0** | ***1.2(0.9-1.5)*** |
| **Number of physician visits 6-10** | **vs** | **0-5** | ***1.1(0.9-1.3)*** |
| **11-15** | **vs** | **0-5** | ***1.0(0.8-1.3)*** |
| **>15** | **vs** | **0-5** | ***1.1(0.9-1.4)*** |
| **Hospitalization at baseline** | | | ***1.2(1.0-1.5)**** |
| **Long-term care stay at baseline** | | | ***0.6(0.5-0.7)**** |
| **Fracture at baseline** | | | ***0.9(0.7-1.2)*** |
| **Dual-energy X-ray absorptiometry at baseline** | | | ***0.8(0.7-0.9)**** |
| **Internal medicine physician visit at baseline** | | | ***1.0(0.8-1.2)*** |
| **Family practice physician visit at baseline** | | | ***0.9(0.8-1.1)*** |
| **Oncologist visit at baseline** | | | ***1.0(0.8-1.2)*** |
| **Rheumatologist or endocrinologist visit at baseline** | | | ***0.8(0.7-1.0)**** |
| **Osteoporosis** | | | ***0.9(0.7-1.0)**** |
| **Proton pump inhibitors** | | | ***0.8(0.7-1.0)**** |
| ^a^Discontinuation definition I: Totally discontinued all anti-osteoporosis drugs as of the end of follow up.  ^b^Adjusted for all factors listed in the table, and urban/rural residency, entering Medicare part D coverage gap at baseline, glucocorticoid-related and fall-related (predisposing to falls) conditions, diabetes, chronic kidney disease, depressive illness, acute myocardial infarction, other heart disease, metabolic bone disease, cancer, anticonvulsants, antidepressants, antipsychotics, antihypertensives, lipid-lowering drugs, non-steroidal anti-inflammatory drugs, steroids, H2-receptor blockers, hormone replacement therapy, thiazolidinediones,and aromatase inhibitors;  *p<=0.05 | | | |
